# Problematic Digital Behaviors, Procrastination, and Psychological Well-Being: An Integrative Explanatory Model of Happiness in Young Adults

**DOI:** 10.31083/AP47250

**Published:** 2026-04-21

**Authors:** Marc Carrasco-Reig, Irene López-Palanca, Manuel Martí-Vilar, Francisco González-Sala, Javier Esparza-Reig

**Affiliations:** ^1^Department of Psychology, Universidad Europea de Valencia, 46010 Valencia, Spain; ^2^Department of Basic Psychology, Universitat de València, 46010 Valencia, Spain; ^3^Department of Developmental and Educational Psychology, Universitat de València, 46010 Valencia, Spain

**Keywords:** models, theoretical, behavior, addictive, internet use, sexual behaviour, procrastination, psychological well-being, happiness

## Abstract

**Background:**

In recent years, the emergence and development of new technologies have led to an increase in problematic digital behaviors, which have been associated with lower levels of subjective well-being and reduced perceptions of happiness among young people.

**Methods:**

This cross-sectional study employed a non-experimental, descriptive, and correlational design. The aim was to examine the relationships among problematic digital behaviors, maladaptive coping, and subjective well-being, in order to develop an integrative explanatory model. Participants completed a series of online questionnaires assessing the variables of interest. A path analysis was conducted to test the hypothesized model.

**Results:**

The sample consisted of 763 young individuals aged between 18 and 30 years, with a mean age of M = 21.4 (SD = 2.76), and 66.7% were women. Results indicated that problematic internet use was positively associated with problematic pornography use, gaming disorder, and compulsive buying disorder, explaining 12%, 7%, and 13% of their variance, respectively. Moreover, all four behaviors exhibited negative relationships with psychological well-being, with procrastination acting as a mediating variable. The model accounted for 23% of the variance in procrastination and 19% in psychological well-being. Finally, psychological well-being was positively and significantly associated with happiness, explaining 46% of its variance.

**Conclusions:**

The proposed theoretical model demonstrated good fit across all goodness-of-fit indices, highlighting the importance of understanding the role of emerging problematic digital behaviors and their impact on well-being and happiness. These findings support the promotion of self-regulation strategies and healthy digital habits.

## Main Points

- This study develops an integrative and transdiagnostic explanatory model connecting problematic digital behaviors, procrastination, psychological well-being, and happiness in young adults.

- It provides novel empirical evidence on how different forms of behavioral addictions (problematic internet use, compulsive buying, problematic pornography use, and gaming disorder) interact and influence mental health indicators.

- The study employs path analysis with a robust methodological design and demonstrates an optimal model fit, highlighting the mediating role of procrastination in the link between digital addictions and psychological well-being.

- Our findings contribute to the prevention and promotion of mental health, offering a new theoretical framework for understanding digital behavioral addictions in contemporary societies.

## 1. Introduction

The widespread use of the internet has transformed access to information, entertainment, and services, offering substantial benefits in terms of speed and convenience. However, these digital practices have also been associated with the emergence of maladaptive behaviors [1]. In particular, excessive internet use has been consistently linked to a range of mental health problems, including anxiety, depression, and psychological distress [2]. Such behaviors may function as dysfunctional coping mechanisms in response to negative emotional states, thereby suggesting a potentially bidirectional relationship between problematic internet use and psychological disorders [3].

One such maladaptive behavior is procrastination, defined as the voluntary delay of intended tasks despite anticipating negative consequences [4]. Procrastination has been linked to impairments in emotional regulation, increased stress and anxiety, and lower perceived well-being [5]. Consequently, this pattern of behavior entails reduced task engagement and diminished subjective well-being, ultimately affecting individuals’ perceived happiness and life satisfaction [6].

The digital environment facilitates procrastination through the constant availability of multiple sources of immediate gratification, which interferes with the pursuit of personal goals [7]. Behaviors such as excessive pornography use, uncontrolled browsing, problematic gaming, and compulsive buying share mechanisms related to emotional avoidance and the pursuit of escape, and they exert detrimental effects on mental health—particularly when these digital contents operate as immediate reinforcers in contrast to tasks requiring sustained cognitive effort [8, 9].

The interaction between emotional avoidance and the pursuit of immediate gratification facilitates the emergence of uncontrolled behaviors and behavioral dysregulation [10, 11, 12]. Problematic Internet Use (PIU) is defined as a pattern of internet use characterized by a loss of control, compulsive engagement, and significant interference with daily functioning [13]. When procrastination is examined in relation to PIU, a consistent association emerges: PIU appears to function both as a trigger for and a maintaining factor of procrastinatory behavior [9].

Problematic Pornography Use (PPU) has been identified as a digital behavior associated with negative mental health outcomes, with higher prevalence rates and greater severity levels observed among men [14]. This compulsive pattern of consumption has been associated with difficulties in emotional regulation, heightened anxiety, depressive symptoms, and lower life satisfaction [15]. Moreover, PPU may operate as an emotional-avoidance strategy, fostering procrastination by diverting attention away from important responsibilities [16].

Problematic gaming behavior (Gaming Disorder, GD) is also associated with elevated levels of psychological symptoms and increased procrastination. The highly stimulating nature of video games facilitates the displacement of personally relevant activities, intensifying the perceived loss of control and negatively affecting self-esteem [17, 18, 19]. A meta-analysis by Stevens *et al*. [20] showed that male gender constitutes a risk factor.

Compulsive online buying (Compulsive Buying Disorder, CBD), particularly when linked to impulsivity, is associated with emotional avoidance and the pursuit of immediate gratification. This pattern reflects dysfunctional coping strategies and deficits in self-control, thereby fostering procrastination [21, 22], and is observed more frequently in women [23].

The literature distinguishes between psychological well-being and happiness, the latter understood as an experience linked to life satisfaction. Psychological well-being encompasses relatively stable aspects related to personal development, self-determination, and a sense of purpose [24], whereas happiness is conceptualized as a more immediate expression of subjective well-being, derived from an individual’s appraisal of their life and everyday emotional states [25, 26].

From this distinction, it is coherent to position happiness as a later outcome within the model, as it represents a more proximal and experiential manifestation of positive psychological functioning. Moreover, continuous exposure to rewarding stimuli (as occurs in the context of the problematic digital behaviors described above) may alter these foundational components of well-being and influence the subjective experience of happiness [27].

The literature indicates that the problematic digital behaviors examined in this study (PIU, PPU, GD, and CBD) share processes of emotional dysregulation, the pursuit of immediate gratification, and avoidance [27, 28]. Within these mechanisms, procrastination is particularly relevant because it represents a deficient self-regulation pattern that integrates cognitive, motivational, and emotional components, providing a contextual understanding of the shift toward more immediately rewarding and less effortful digital activities.

Within the framework of the Interaction of Person–Affect–Cognition–Execution (I-PACE) model [27], procrastination functions as a coping and execution mechanism through which personal and affective factors may relate to the emergence of maladaptive digital behaviors. Accordingly, PIU, PPU, GD, and CBD can be interpreted as behavioral manifestations within the execution phase of the model. Although this study does not directly assess the dispositional factors proposed by I-PACE, the model provides conceptual coherence and clarifies the logical sequence between self-regulatory processes and the digital behaviors analyzed.

Following this logic, the theoretical model of the study is organized sequentially: PIU operates as a general pattern of digital dysregulation; PPU, GD, and CBD represent specific behaviors associated with impulsivity, immediate reinforcement, and avoidance; procrastination serves as a mediator by explaining how these behaviors erode well-being through task postponement; and finally, psychological well-being and happiness constitute the subjective outcomes.

Based on this rationale, the overarching objective of the study is to develop and test a novel explanatory model that accounts for the interrelations among different problematic behaviors (PIU, PPU, CBD, and GD) and their impact on psychological well-being and happiness, considering procrastination as a mediating variable. This general objective is further broken down into three specific objectives: Specific Objective 1 aims to examine whether PIU is associated with the other three problematic behaviors evaluated (PPU, CBD, and GD); Specific Objective 2 seeks to analyze whether the four problematic behaviors (PIU, PPU, CBD, and GD) are related to psychological well-being, using procrastination as a mediating variable of these relationships; and Specific Objective 3 aims to evaluate whether psychological well-being is positively associated with perceived happiness.

**Fig. 1.**
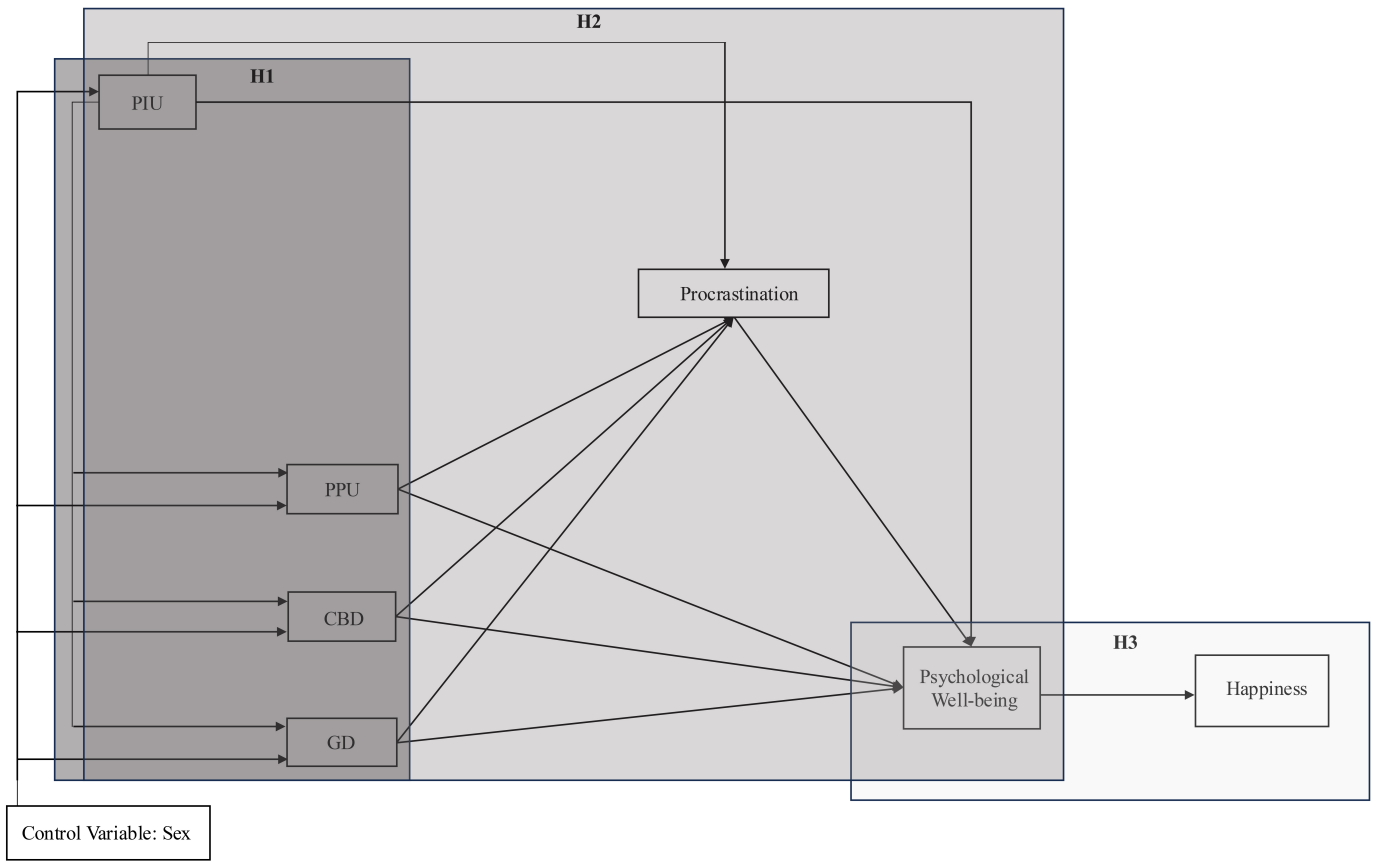
**Hypothesized model of the relationships between problematic digital behaviors, procrastination, psychological well-being, and happiness**. PIU, Problematic Internet Use; PPU, Problematic Pornography Use; CBD, Compulsive Buying Disorder; GD, Gaming Disorder (Videogame); H1, Hypothesis 1; H2, Hypothesis 2; H3, Hypothesis 3.

Based on these objectives, a series of hypotheses are proposed: Hypothesis 1 (H1) posits that PIU will be associated with the other three problematic behaviors (PPU, CBD, and GD). Hypothesis 2 (H2) proposes that the four problematic behaviors (PIU, PPU, CBD, and GD) will be inversely related to psychological well-being, with procrastination acting as a mediating variable in these relationships. Finally, Hypothesis 3 (H3) posits that psychological well-being will be positively associated with happiness. All proposed hypotheses are illustrated in the theoretical model shown in Fig. 1.

## 2. Materials and Methods

### 2.1 Procedure

A cross-sectional quantitative study was conducted, following a non-experimental, descriptive, and correlational design. For data collection, non-probabilistic sampling techniques were employed, specifically convenience and snowball sampling. Data were collected between February 2023 and April 2024 through an online survey that provided participants access to a battery of questions related to the research. Participants were recruited through both online and in-person sampling procedures. Specifically, recruitment was conducted via social media platforms and instant messaging applications, as well as through visits to university classrooms to directly contact students and facilitate their participation in the study. All participants took part voluntarily and without any form of compensation, with the only eligibility criterion applied was that participants were between 18 and 30 years of age. Prior to completing the questionnaire, all participants read and provided informed consent, which detailed the study’s objectives and guaranteed anonymity and confidentiality of the data provided, as well as stipulating that the questionnaire could only be completed once.

### 2.2 Instruments

A battery of validated psychological scales in Spanish was employed, in addition to a set of ad hoc sociodemographic questions designed to describe the main characteristics of the sample. For most of the scales used, no additional adaptations or sample-specific pilot testing were conducted, as validated versions were already available. The only exception was the Multicage-Information and Communications Technologies (ICT), for which the adaptation and validation procedures are described below. The psychological scales selected to measure the study variables were as follows:

The Multicage-TIC [29] was used to assess the two variables related to problematic use of new technologies (ICT), specifically Internet use and video gaming. This scale comprises five factors or subscales, each focused on exploring a specific problem, including PIU, GD, problematic smartphone use, social media use, and instant messaging use. It consists of 20 items originally measured using a dichotomous scale (yes/no); however, an adapted Likert-type version with six response anchors was employed. The new Likert-type model employed demonstrated optimal fit in the confirmatory factor analysis, with values of χ^2^/df = 2.88, Comparative Fit Index (CFI) = 0.99, Tucker–Lewis Index (TLI) = 0.98, and Root Mean Square Error of Approximation (RMSEA) = 0.049. Reliability analyses for the two subscales used yielded values of α = 0.77 and ω = 0.78 for PIU, and α = 0.86 and ω = 0.86 for GD.

To assess the presence of CBD, the Bergen Shopping Addiction Scale (BSAS) [30] was applied in its Spanish-adapted version [31]. This scale is designed to detect potential problematic shopping patterns, both in-person and online. The abbreviated version of the instrument consists of a single factor composed of 7 items, measured using a five-point Likert-type scale ranging from “strongly disagree” to “strongly agree”. In the study sample, Cronbach’s alpha was 0.85 and McDonald’s omega was 0.88.

To measure online PPU in young adults, the sex techno-addiction scale [32] was used. This unidimensional instrument comprises 11 items, rated on a five-point Likert-type scale ranging from “never” to “always”. Reliability analyses yielded α = 0.90 and ω = 0.91 for the scale scores.

To assess procrastination, the General Procrastination Scale [33], adapted into Spanish from the original scale developed by Busko [34], was used. This instrument allows for the measurement of both general and academic procrastination; however, only the general dimension was considered in the present study. The unidimensional scale consists of 13 items, rated on a five-point Likert-type scale ranging from “never” to “always”. In the study sample, the internal consistency of the instrument scores was α = 0.90 and ω = 0.91.

The General Health Questionnaire (GHQ-12) [35], in its Spanish-adapted version [36], was used to assess psychological well-being in participants. This scale consists of 12 items and, depending on interpretations, can be structured factorially in various ways. In this study, however, it was treated as a unidimensional scale, following the approach proposed by González-Romá *et al*. [37]. Reliability analyses yielded α = 0.89 and ω = 0.89.

Finally, to measure happiness, the Spanish version of the Subjective Happiness Scale (SHS) [38], originally developed by Lyubomirsky and Lepper [25], was employed. This unidimensional instrument comprises 4 items and adopts a subjectivist approach to measuring happiness. Items are rated on a seven-point Likert scale. In the study sample, reliability analyses yielded α = 0.79 and ω = 0.81.

### 2.3 Analysis

In a first phase, the distribution and response frequencies for each variable included in the study were examined to extract descriptive statistics for the sample. Additionally, to assess the psychometric properties of the instruments used, confirmatory factor analyses and reliability analyses using Cronbach’s alpha and McDonald’s omega were conducted for all scales and subscales.

Subsequently, a path analysis (PA) was performed to test the hypothesized model presented in Fig. 1. This technique allows for the evaluation of the fit of theoretical models proposing a set of dependency relationships among variables [39]. Sample size recommendations for this type of analysis were followed [40]. For the analysis, the robust maximum likelihood estimation method was used, given the multivariate non-normality detected through Mardia’s coefficient and the Anderson-Darling test. Multicollinearity among the variables employed was also ruled out (see Table 1). For the path analysis, path coefficients are presented, which are analogous to beta coefficients in linear regression [41].

**Table 1.** **Bivariate correlations**.

	1. PIU	2. PPU	3. CBD	4. GD	5. PR	6. WB	7. H
1. PIU	-						
2. PPU	0.05	-					
3. CBD	0.22***	0.07*	-				
4. GD	0.30***	0.16***	0.09*	-			
5. PR	0.42***	0.18***	0.20***	0.24***	-		
6. WB	–0.23***	–0.11**	–0.20***	–0.21***	–0.40***	-	
7. H	–0.16***	–0.09*	–0.13***	–0.18***	–0.29***	0.68***	-

GD, Gaming Disorder (Videogame); PR, Procrastination; WB, Psychological Well-being; H, Happiness; *p < 0.05, **p < 0.01, ***p < 0.001.

Although all goodness-of-fit indices resulting from the analyses were examined to identify potential errors or misfits, only the values for CFI, Goodness of Fit Index (GFI), RMSEA, NFI (Normed Fit Index), and SRMR (Standardized Root Mean Square Residual) their robust versions are presented. The following cut-off points were considered for these indices: values above 0.95 were regarded as indicating optimal fit for CFI, GFI, and NFI [42, 43]. Additionally, for the RMSEA and SRMR, values below 0.06 and 0.05, respectively, were considered indicative of optimal model fit [42, 43], and its 95% confidence interval and statistical significance were reported, allowing a more precise evaluation of the model’s discrepancy relative to the population.

The χ^2^/df ratio was used to evaluate the overall model fit. Because the χ^2^ statistic is highly sensitive to sample size—often yielding statistically significant values even when the model fits well in large samples—the χ^2^/df ratio provides a more stable indicator of fit. Ratios between 2 and 3 are typically considered indicative of good fit [44]. It should be noted that this index must always be interpreted jointly with the CFI, TLI, and RMSEA, thereby offering a more robust and balanced assessment of model fit.

Finally, mean comparisons were conducted using the Mann–Whitney U test to examine sex differences in the four continuous variables related to problematic digital behaviors. Normality assumptions were assessed using the Shapiro–Wilk test. Effect sizes were estimated using Cohen’s d, following the conventional thresholds of 0.20, 0.50, and 0.80 for small, medium, and large effects, respectively [45], as well as the additional benchmark for very large effects (d = 1.20) [46]. Statistical analyses were conducted using the R statistical software (version 4.4.3; R Foundation for Statistical Computing, Vienna, Austria) with the lavaan package [47].

## 3. Results

The sample consisted of 875 participants, from which a subsample of 763 individuals was selected for the present study based on the age-related inclusion criteria. The age range was set between 18 and 30 years, with a mean age of 21.4 years (SD = 2.76). Regarding biological sex, 66.7% of the participants were women (n = 509), and 33.3% were men (n = 254). Most participants (90.7%) were Spanish nationals. Concerning marital status, 51.5% (n = 393) were single, 48.1% (n = 367) were in a relationship, and 0.4% (n = 3) were married.

Regarding sociocultural level, frequencies were analyzed for educational attainment and employment status. In terms of education, 0.7% (n = 5) had completed only primary education, 1.4% (n = 11) had completed compulsory secondary education, 19.4% (n = 148) held a high school diploma, 67.8% (n = 517) had a university degree, 10.2% (n = 78) had postgraduate studies, and 0.5% (n = 4) held a doctoral degree. Regarding employment status, 61.6% (n = 470) were full-time students, 22.5% (n = 172) were combining work and studies, 13.6% (n = 104) were employed full-time, and 2.3% (n = 17) were unemployed or in other circumstances.

**Fig. 2.**
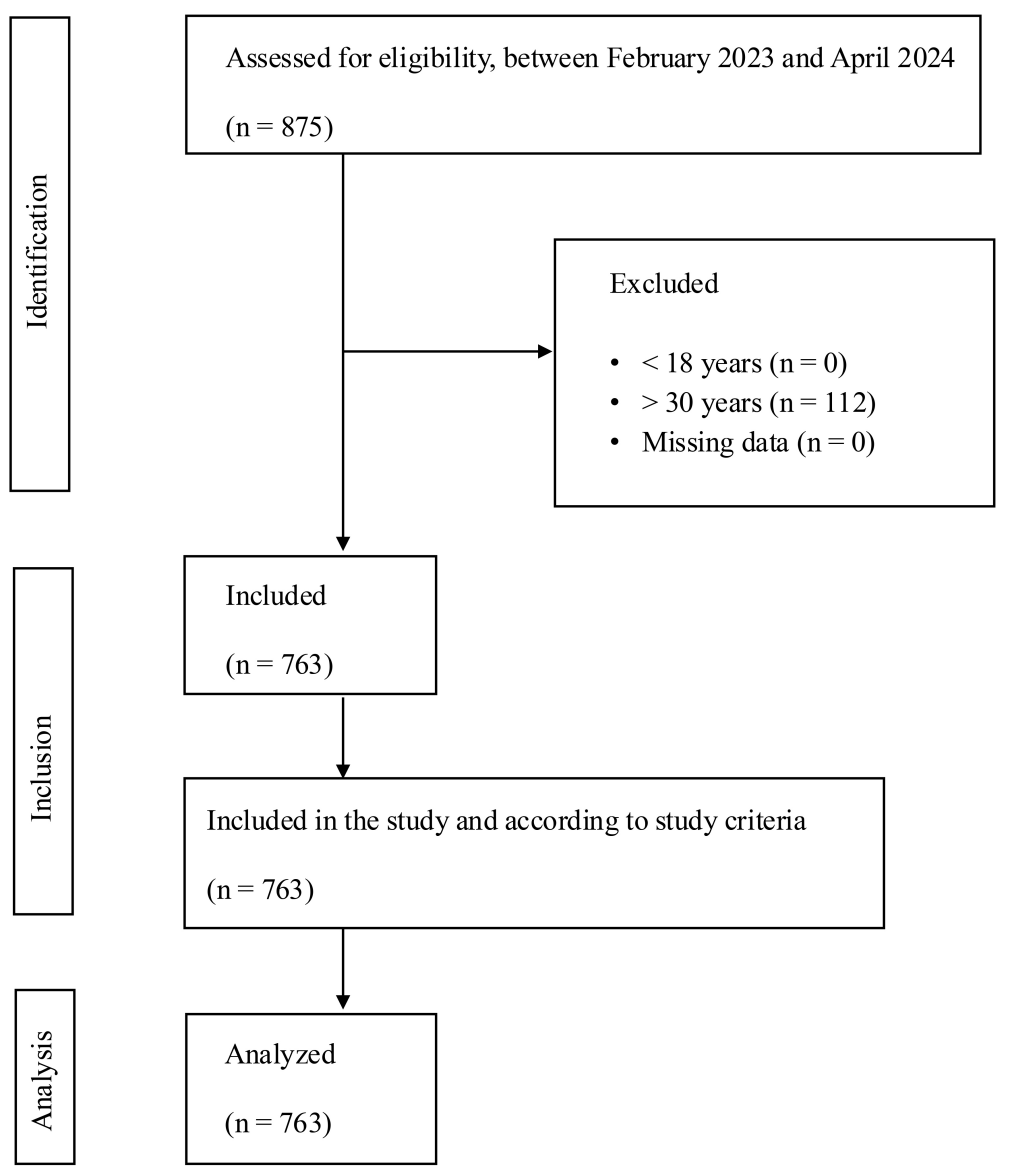
**Flow diagram of the participant selection process**.

Participant selection was conducted in accordance with predefined age-based inclusion criteria (see Fig. 2). There were no missing values for any of the variables measured and analyzed. It should be noted that, since the survey was distributed electronically, it was not possible to account for all individuals who were contacted and declined to participate in the study.

Prior to hypothesis testing, after verifying that the collected variables did not follow a normal distribution using Mardia’s coefficient and the Anderson-Darling test, a Pearson correlation analysis was conducted among all variables to rule out the presence of multicollinearity. The results of these correlations are presented in Table 1, showing that no high correlations were observed that could indicate potential multicollinearity [39].

**Fig. 3.**
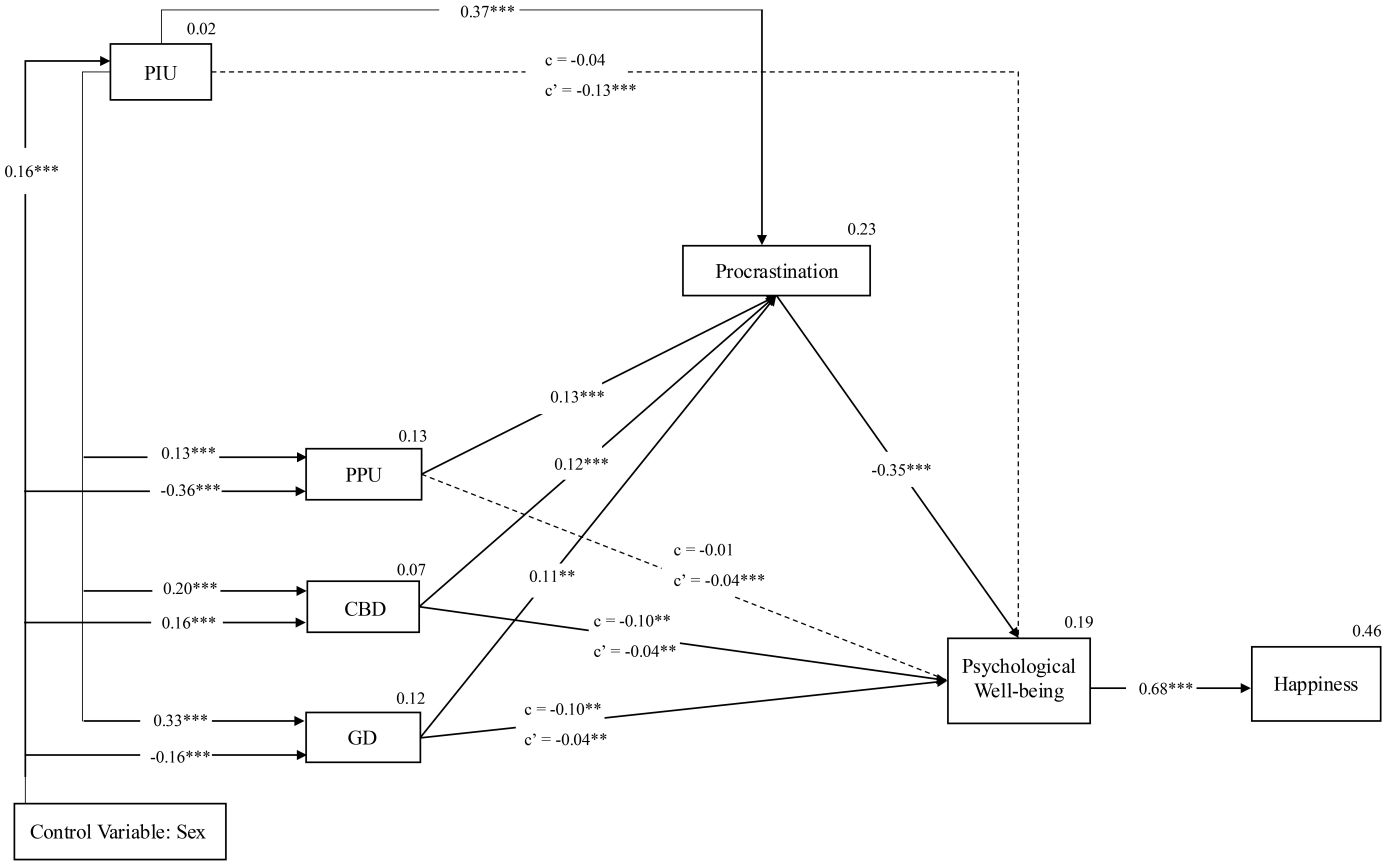
**Hypothesized model with standardized path coefficients**. In the figure, dotted lines represent direct relationships that were not statistically significant. The arrows indicate the direction of the paths specified in the model. Since the data are cross-sectional, these paths should be interpreted as directional associations rather than predictive or causal effects. c = direct effects; c’ = indirect effects; solid lines = significant relationships; dashed lines = non-significant relationships; **p < 0.01, ***p < 0.001.

After conducting the PA depicted in Fig. 3, a statistically significant relationship was first observed between sex and all problematic behaviors analyzed. Specifically, the “male” category was associated with higher scores in PPU (β = –0.36, *p* < 0.001) and GD (β = –0.16, *p* < 0.001), whereas the “female” category was associated with higher scores in PIU (β = 0.16, *p* < 0.001) and CBD (β = 0.16, *p* < 0.001).

Subsequently, mean comparisons were conducted using the Mann–Whitney U test due to deviations from normality in all variables (Shapiro–Wilk, *p* < 0.001), and effect sizes were calculated using Cohen’s d. Results revealed significant sex differences across all problematic digital behaviors (*p* < 0.001), with higher means observed in men for PPU (16.72 vs. 12.77; *d* = 1.15) and GD (8.55 vs. 7.19; *d* = 0.45), and in women for PIU (15.20 vs. 13.81; *d* = 0.33) and CBD (29.95 vs. 22.72; *d* = 0.41). The magnitude of the effects indicates a very large effect for PPU, whereas the remaining effects are of medium size.

When analyzing the relationship among all problematic behaviors, with PIU as the independent variable (IV) and the others as dependent variables (DV), all relationships were statistically significant. In particular, the strongest relationship was observed with GD (β = 0.33, *p* < 0.001), followed by CBD (β = 0.20, *p* < 0.001), and lastly PPU (β = 0.13, *p* < 0.001). The model, as indicated by *R*^2^, explained 12%, 7%, and 13% of the variance of the DVs, respectively.

Second, a series of mediation models were analyzed, with the different problematic behaviors as IVs, psychological well-being as the DV, and procrastination as a mediator for each of these relationships. In the cases of GD and CBD, the results showed partial mediation, as both the direct and indirect effects were statistically significant; in both instances, the relationship between the problematic behaviors and psychological well-being remained significant, and the inclusion of procrastination in the model was associated with an increased magnitude of the estimated effect, with the total effect size being significant in both cases (GD: β = –0.14, *p* < 0.001; CBD: β = –0.14, *p* < 0.001). In both cases, the negative effect of the problematic behaviors on psychological well-being was amplified when including the effect of procrastination.

On the other hand, in the case of PIU and PPU, the direct effect of each of these variables was not statistically significant, whereas the indirect effect through procrastination as a mediator was significant. These results are consistent with a pattern of full mediation, as the association between the independent variables and the dependent variable was observed only through the mediator. In both cases, the total effect was statistically significant (PIU: β = –0.15, *p* < 0.001; PPU: β = –0.05, *p* < 0.001). The model, as indicated by *R*^2^, explained 23% of the variance in procrastination and 19% of the variance in psychological well-being. The detailed indices obtained can be seen in Fig. 3.

Finally, when analyzing the relationship between psychological well-being as the IV and happiness as the DV, a positive and statistically significant association was found (β = 0.68, *p* < 0.001), explaining 46% of the variance in happiness. Overall, the theoretical model demonstrated an adequate fit to the data. The χ^2^ statistic indicated χ^2^(9) = 26.10, *p* = 0.002, and the χ^2^/df ratio was 2.90, a value typically considered indicative of good fit [44]. Additional goodness-of-fit indices confirmed optimal model fit: CFI = 0.994, TLI = 0.982, NFI = 0.973, RMSEA = 0.031 (95% CI [0.028, 0.073], *p* = 0.463), and SRMR = 0.050.

## 4. Discussion

The overall aim of the present research was to develop an explanatory model defining the interrelationships among specific problematic behaviors and their impact on psychological well-being and happiness, considering procrastination as a mediating variable. To this end, three hypotheses were proposed, all of which were empirically supported by the results obtained.

Regarding H1, it was proposed that PIU would be associated with PPU [27], CBD [48], and GD [49]. The results were consistent with this proposition, as all relationships were statistically significant. The PIU model framework posits that problematic digital behaviors are influenced by individual vulnerabilities and dysfunctional cognitive processes, which act as transdiagnostic factors that amplify their impact on psychological well-being and happiness [27]. In this regard, the significant associations of PIU with PPU, CBD, and GD supports the presence of strong associations among these problematic online behaviors, which may help explain the comorbidity observed in the data [48, 49].

Regarding H2, it was postulated that the four problematic behaviors (PIU, PPU, CBD, and GD) would be inversely associated with psychological well-being, with procrastination exerting a mediating these relationships [6, 8, 9]. The analyses yielded results partially consistent with the proposed hypothesis: they showed that GD and CBD exhibited both direct and indirect negative associations with well-being, whereas PIU and PPU were related to well-being only indirectly through procrastination.

This difference in relationship patterns may be explained by the nature of each problematic behavior. For GD and CBD, their direct association with lower psychological well-being is consistent with previous studies showing that these behaviors tend to produce immediate and concrete consequences in daily life, such as deterioration in social relationships, reduced academic or occupational performance, financial strain, family or interpersonal conflict, and substantial time loss, all of which directly undermine indicators of psychological well-being [48, 49, 50].

Conversely, PIU and PPU exhibit an indirect association with well-being, as suggested by theoretical models linking PIU to deficient self-regulatory processes [9, 27]. In this regard, procrastination plays a key role as both a mediating mechanism: these behaviors primarily exert their impact indirectly through procrastination as an avoidance strategy, delaying academic or occupational tasks and increasing feelings of guilt, stress, and emotional distress [6, 8]. Moreover, the mean comparisons and effect sizes suggest sex-specific patterns: men scored higher on PPU and GD, whereas women scored higher on PIU and CBD. These findings support the notion that certain behaviors are more directly associated with well-being, while others primarily exhibit links mediated through procrastination.

Taken together, these results suggest that whereas GD and CBD exhibit a more direct relationship with psychological well-being, PIU and PPU are primarily associated through a pattern of ineffective procrastination. This pattern not only modulates the magnitude of the impact of these behaviors on well-being but also represents a critical factor in understanding why some problematic behaviors show mediated associations, whereas others are related more directly.

Finally, H3 proposed that psychological well-being would be positively associated with happiness. Statistically significant and positive results were obtained, which are supported by various studies. According to Huppert [26], psychological well-being constitutes a robust predictor of subjective happiness, as it encompasses dimensions such as purpose in life, self-acceptance, positive relationships, and autonomy, which are consistently associated with higher perceived happiness. Moreover, recent research indicates that low levels of psychological well-being tend to correlate with lower happiness, whereas high levels of well-being act as a protective factor against negative emotional symptoms and promote a more satisfying life [51].

However, happiness is a multidimensional construct that may be influenced by factors beyond psychological well-being, such as social support, personality traits, or socioeconomic circumstances [52]. These variables may interact bidirectionally with problematic behaviors, either amplifying or attenuating their impact on happiness. Nonetheless, given the cross-sectional nature of this study, it is not possible to empirically assess this potential bidirectionality.

Overall, the findings allow us to conclude that the results align with the proposed explanatory model. This not only means that the expected relationships were statistically significant, but also that the observed pattern is consistent with the theoretical mechanisms proposed in current models of problematic online behaviors and psychological well-being.

Regarding the study’s limitations, the first pertains to the type of sampling employed, which was non-probabilistic. While such procedures are common in applied research in similar contexts, they may limit the generalizability of the results. This strategy was chosen due to the logistical and accessibility challenges associated with implementing probabilistic sampling methods in studies of this nature [53]. Nevertheless, future research should consider the implementation of random or stratified sampling procedures whenever possible, in order to enhance the external validity of the findings.

A second limitation of the study is its exclusive reliance on self-report measures, which may introduce response biases, such as social desirability or common method bias. Future research could combine self-reports with complementary methods, such as external assessments or physiological data, to mitigate this type of bias.

The third limitation is related to the nature of the selected sample. As the sample was drawn from the general population, it is possible that participants with subclinical symptoms or specific problematic behaviors were not sufficiently represented, which could affect the sensitivity of the model. Moreover, the sample was composed primarily of Spanish-speaking young adults with a high educational level, which limits the generalizability of the findings to other populations. Future studies could test the model in clinical samples with distinct characteristics and in more diverse populations, both in terms of age, educational level, and sociocultural context, with the aim of evaluating its validity, generalizability, and applicability across broader and more targeted contexts.

The fourth limitation concerns the methodology employed. Analyses conducted through PA constitute an appropriate strategy to describe the consequences of a series of causal hypotheses within a system of relationships among variables [54]. However, this approach does not allow for definitive causal inferences; rather, it helps to select or infer among existing causal hypotheses [55]. Consequently, it is recommended that future research incorporate more robust statistical methods, such as structural equation modeling, which allow for the inclusion of latent variables and the simultaneous analysis of complex relationships among multiple constructs. Likewise, the use of controlled experimental designs could help confirm the proposed hypotheses and strengthen the internal validity of the results. In this regard, Kline [56] caution that causal inferences in PA should be made carefully, as different explanatory models may present equally adequate fit to empirical data.

In line with this, another limitation concerns the fact that some of the relationships specified in the model exhibited small effect sizes, warranting cautious interpretation of the results. Future research should replicate this model in larger and more diverse samples to determine the stability and generalizability of the findings.

Finally, a fifth limitation is the absence of external validation using an independent sample, which prevents fully ensuring the stability, robustness, and generalizability of the model. Consequently, it is recommended that future research conduct external validation using new samples, preferably more heterogeneous ones, in order to confirm the replicability of the proposed structure and strengthen the robustness of the findings.

## 5. Conclusions

This study provides evidence on how various problematic digital behaviors interrelate and affect psychological well-being and, consequently, happiness. The findings indicate that higher PIU scores are associated with higher scores in these problematic digital behaviors, while procrastination functions as a mediator, channeling part of its impact on well-being. In turn, psychological well-being is positively associated with happiness, highlighting the importance of promoting self-regulation strategies and healthy digital habits.

From a theoretical perspective, the developed model contributes to refining the understanding of problematic online behaviors, showing that they are not isolated phenomena but interconnected behaviors that share common psychological processes. The finding that PIU acts as a transdiagnostic risk factor supports the need for transdiagnostic conceptual approaches [27], in which individual vulnerabilities and self-regulatory deficits are central elements in explaining how problematic digital behaviors jointly affect psychological well-being and happiness, highlighting the concomitant emergence of these problems and their impact on mental health indicators. Additionally, the mediating role of procrastination provides a novel nuance, indicating that time management and coping strategies play a decisive role in the relationship between problematic digital behaviors and well-being.

From a practical perspective, the findings underscore the importance of identifying usage patterns and habits associated with greater psychological well-being, with the aim of enhancing them and promoting a better quality of life. In this regard, interventions could include specific components, such as effective time management programs, cognitive-behavioral therapy (CBT) modules targeting procrastination, and training in self-regulation skills.

In summary, this study proposes a new explanatory, integrative, and transdiagnostic model. The identification of both protective and risk patterns represents a significant step toward the consolidation of more robust conceptual frameworks and the development of strategies that, rather than focusing solely on the reduction of distress, also aim to enhance well-being and quality of life.

## Availability of Data and Materials

The datasets generated or analyzed during the present study are not publicly available due to ethical and legal restrictions. Most of the relevant data supporting the findings of this study are included in the article. Additional data can be requested from the corresponding author upon reasonable request.
